# Optimal management of waste loading into a river system with nonpoint source pollutants

**Published:** 2004-08-01

**Authors:** Toshihiko Kawachi, Shigeya Maeda

**Affiliations:** Graduate School of Agricultural Science, Kyoto University, Kitashirakawa Oiwake-cho, Sakyo-ku, Kyoto 606-8502

**Keywords:** Optimization, wasteload allocation, river water quality management, nonpoint source, uncertainty, *ε*-constraint method

## Abstract

An optimization model, fit for practical use, to allocate COD (Chemical Oxygen Demand)-based wasteload into a river system among outfalls is developed within the framework of robust optimization (RO). Nonpoint source COD loading, estimated based on the unit loading factor to be assumed known, is treated as uncontrollable one. The total amount of expected allowable COD load from point sources is then, under all possible scenarios of uncertain input information, maximized while satisfying the constraints on in-stream COD and DO (Dissolved Oxygen) transport, effluent standards and river water quality standards. Advantage of the *ε*-RO model using the *ε*-constraint method for optimization practice is brought to light from theoretical and practical aspects, in comparison with the conventional RO model resorting to the Lagrangian method. Solving a simple hypothetical example problem, it is demonstrated that the model developed is competent for successfully generating noninferior and robust solutions on optimal COD load allocation.

## Introduction

Pollution control or abatement is an emergent need in many rivers of the world. A variety of decision-support models that provide options or alternatives for strategic management of river water quality have been proposed. Recent advanced researches^e.g.^ 1)–4) have been successful in modeling the uncertain nature of hydrological/hydraulic riverine environment and treating the river water quality management problem as a multiobjective optimization problem. Mostly they include objective or constraint, expressed in terms of cost, or cost and benefit, to minimize an outlay for sewage treatment or pollution abatement. Such a cost/benefit-based approach is rather suitable for design and operation of sewage treatment plants, and may produce less robust solutions since it is not free of mobility and uncertainty in market analysis of costs and benefits. The optimization models that the authors have presented5)–7) are cost/benefit-free, and can serve as master supervisory optimization models that provide normative effluent limits with that distributed local dischargers in a river basin must comply. The models allocate or reallocate an overall allowable BOD (Biochemical Oxygen Demand) load among particular loading points or existing outfalls in a river under hydro-environmental uncertainties, and therefore decide maximum allowable loads from controllable sources (e.g., industrial and sewage plants) or point sources (PSs). However, they lack for considering difficult-to-control or uncontrollable pollutant loads from diffused or nonpoint sources (NPSs) like agricultural land, forest or city into a river.

This paper presents a sophisticated optimization model fit for practical use, making significant improvements over the previous works.5)–7) Waste loadings from both PSs and NPSs are considered. As a water quality indicator, COD (Chemical Oxygen Demand) is employed in lieu of BOD since pollutant loads from NPSs are commonly predicted in terms of COD-based unit loading factors. More practical way for generating scenarios that describe hydro-environmental uncertainties in a stochastic sense is also shown.

The model presently developed is also within the framework of robust optimization,8) and, as in the latest work,7) employs the *ε*-constraint method9) in order to reduce a vector RO problem to a scalar one. Advantages of using the *ε*-constraint method are more clearly brought to light from theoretical and practical aspects. With a simple hypothetical example problem, it is demonstrated that the *ε*-RO model is capable of effectively producing significant options or alternatives for river water quality management.

## COD and DO transport equations

Suppose that, due to gravity, wastewaters issued from interspersed PSs in a river basin of interest come up to outfalls (i.e., loading points) in the river. The variations of COD and DO (Dissolved Oxygen) along the river are assumed to be represented by the following one-dimensional steady-state transport equations, respectively.

[1] $$Q\frac{dL}{dx}-\frac{d}{dx}\left(AD_{x}\frac{dL}{dx}\right)+AK_{1}L+\left({\bar{q}}^{p}+{\bar{q}}^{np}\right)L-{\bar{q}}^{p}L^{p}={\bar{q}}^{np}L^{np},$$

[2] $$Q\frac{dC}{dx}-\frac{d}{dx}\left(AD_{x}\frac{dC}{dx}\right)+AK_{1}L-AK_{2}\left(C_{S}-C\right)+\left({\bar{q}}^{p}+{\bar{q}}^{np}\right)C=0,$$

where the superscripts *p* and *np* denote point and nonpoint sources, respectively, *x* = horizontal distance along the river (m), *Q* = cross-sectional discharge (m^3^·s^−1^), *A* = cross-sectional area (m^2^), *L* and *C* = concentrations of COD and DO in main stream water, respectively (mg·L^−1^), *L^p^* and *L^np^* = COD concentrations in PS- and NPS-born wastewaters, respectively (mg·L^−1^), *q̄^p^* and *q̄^np^* = lateral discharges per unit width issued from PSs and NPSs, respectively (m^2^·s^−1^), *D_x_* = longitudinal dispersion coefficient (m^2^·s^−1^), *K*_1_ = deoxygenation coefficient (s^−1^), *K*_2_ = reaeration coefficient (s^−1^), and *C_S_* = saturation value of DO (mg·L^−1^). Note that removal of COD by settling and supply of oxygen by lateral discharge are neglected in the transport equations. In developing the optimization model, *L^p^* is taken as a decision variable to be controlled, while *L^np^* as a given, uncontrollable variable.

## Scenario generation

In RO model, scenario-based description of problem input data is made to express their uncertainties in a stochastic sense. For the current problem, considering over-the-year hydro-environmental uncertainties on a monthly basis can provide plausible scenarios. Accordingly the probability of occurrence of each scenario is given as a ratio of the number of days that the corresponding month contains to the year-round number of days. A set of twelve scenarios, which occur probabilistically and each of which comprises monthly measured data of uncertain input parameters, can thus be generated. The basic uncertain parameters to be considered are upstream boundary values of discharge $Q_{i}^{b}$, COD concentration $L_{i}^{b}$, DO concentration $C_{i}^{b}$, and water temperature *T* (°C) where the subscript *i* varies from 1 to the number of upstream boundaries. Also, downstream boundary value of water depth $h_{i}^{b}$, where the subscript *i* ranges from 1 to the number of downstream boundaries, can be a significant scenario component. Furthermore, in this study, discharge from nonpoint sources $q_{j}^{np}$ takes a new component in the scenario. The parameters *D_x_*, *C_S_*, *K*_1_ and *K*_2_ can be estimated by using appropriate functional relations expressed in terms of basic hydraulic variables such as *Q*, *h* and *T*, or be identified if necessary field data are available. Therefore, these can substantially be treated as uncertain input parameters.

## Multiobjective programming problem

Based on the framework of RO, a four-objective optimization problem can be represented in a form of vector optimization as

[3] $$\text{Minimize }\;\;\left\{f_{1}\left(\cdot \right),f_{2}\left(\cdot \right),f_{3}\left(\cdot \right),f_{4}\left(\cdot \right)\right\}$$

subject to

- COD and DO transport equations that are discretized by the finite element method (FEM)10) at all scenarios
  [4] $$E_{s}L_{s}+F_{s}L_{s}^{p}=b_{s},\;G_{s}C_{s}+H_{s}L_{s}=d_{s},\;\;\;\forall s,$$
- Effluent limitation standards for wastewater from PSs at all scenarios
  [5] $$L_{s}^{pl}\leq L_{s}^{p}\leq L_{s}^{pu},\;\;\;\forall s,$$
- Water quality standards (WQSs) at loading points and/or stream junctions in a river at all scenarios
  [6] $$L_{s}^{o}-L_{s}^{ou}=U_{s}^{o+}-U_{s}^{o-},\;\forall s,$$
  [7] $$C_{s}^{ol}-C_{s}^{o}=V_{s}^{o+}-V_{s}^{o-},\;\forall s,$$
- Supplementary constraints at all scenarios
  [8] $$y_{s}^{+}-y_{s}^{-}=x_{s}-\sum_{s}p_{s}x_{s},\;\;\;\forall s,$$
  [9] $$p_{s}(y_{s}^{+}+y_{s}^{-})-w\leq 0,\;\;\;\forall s,$$
- Nonnegativity at all scenarios
  [10] $$\begin{array}{l} L_{s},\;L_{s}^{p},\;L_{s}^{o},\;C_{s},\;C_{s}^{o},\;w,\;y_{s}^{+},\;y_{s}^{-},\\ U_{s}^{o+},\;U_{s}^{o-},\;V_{s}^{o+},\;V_{s}^{o-}\geq 0,\;\;\;\forall s, \end{array}$$

where

[11] $$f_{1}\left(\cdot \right)=-\sum_{s}p_{s}x_{s},$$

[12] $$f_{2}\left(\cdot \right)=\underset{s}{\text{max}}\;\left\{p_{s}\left|x_{s}-\sum_{s}p_{s}x_{s}\right|\right\}=w,$$

[13] $$f_{3}\left(\cdot \right)=\sum_{s}\sum_{i}p_{s}\;\left(U_{is}^{o+}+V_{is}^{o+}\right)$$

[14] $$f_{4}\left(\cdot \right)=\sum_{s}\sum_{i}p_{s}\;\left(U_{is}^{o-}+V_{is}^{o-}\right)$$

[15] $$y_{s}^{+}=\frac{1}{2}\left\{\left|x_{s}-\sum_{s}p_{s}x_{s}\right|+x_{s}-\sum_{s}p_{s}x_{s}\right\},\forall s,$$

[16] $$y_{s}^{-}=\frac{1}{2}\left\{\left|x_{s}-\sum_{s}p_{s}x_{s}\right|-\left(x_{s}-\sum_{s}p_{s}x_{s}\right)\right\},\forall s,$$

the superscripts *u*, *l* and *o* stand for upper limit, lower limit, and loading point and/or stream junction, respectively, the subscript *s* stands for scenario, *p_s_* = probability of scenario *s*, $x_{s}=\sum_{j}q_{j}^{p}L_{js}^{p}$ = total controllable COD loading from PSs into a whole control section of a river at a scenario *s* (g·s^−1^), $q_{j}^{p}$ = given discharge of wastewater related to PSs, injected into a loading point *j* (m^3^·s^−1^), **L***_s_* and **C***_s_* = vectors whose components are COD and DO concentrations in river water at the nodes, respectively, $L_{s}^{p}$ = vector whose component is COD concentration of aggregated wastewater from PSs that is injected into the *j*-th loading points, $L_{js}^{p}\;\left(\text{mg}.{\text{L}}^{-1}\right)$ (decision variable), *E_s_*, *G_s_* and *H_s_* = state matrices obtained from application of the FEM, *F_s_* = matrix associated with $L_{s}^{p}$, **b***_s_* and **d***_s_* = right-hand side vectors, $L_{s}^{pl}$ and $L_{s}^{pu}$ = vectors whose components are upper and lower limits for COD concentration in effluent injected into the *j*-th loading point, $L_{js}^{pl}\;\left(\text{mg}\cdot {\text{L}}^{-1}\right)$ and $L_{js}^{pu}\;\left(\text{mg}\cdot {\text{L}}^{-1}\right)$, respectively, $L_{s}^{ou}$ and $C_{s}^{ol}$ = vectors whose components are limitations of WQSs for COD and DO concentrations, $L_{is}^{ou}$ and $C_{is}^{ol}$, respectively, $U_{s}^{o+}$ and $V_{s}^{o+}$ = vectors of violated deviations of COD and DO concentrations from WQSs and their components are $U_{is}^{o+}\;\left(\text{mg}\cdot {\text{L}}^{-1}\right)$ and $V_{is}^{o+}\;\left(\text{mg}\cdot {\text{L}}^{-1}\right)$, respectively, $U_{s}^{o-}$ and $V_{s}^{o-}$ = vectors of surplus deviations of COD and DO concentrations from WQSs and their components are $U_{is}^{o-}\;\left(\text{mg}\cdot {\text{L}}^{-1}\right)$ and $V_{is}^{o-}\;\left(\text{mg}\cdot {\text{L}}^{-1}\right)$, respectively, and $y_{s}^{+},y_{s}^{-}$ and *w* = supplementary variables. It is assumed that, without any change in quality and quantity, wastewater discharged from any PS in a river basin arrives at a loading point in the river. Thus $\sum_{s}p_{s}q_{j}^{p}L_{js}^{p}$ is a sum of expected COD loads that are discharged from interspersed PSs towards the loading point *j* or injected into the river at that point.

Four component objective functions imply: *f*_1_ is an expected total controllable COD loading with the negative sign; *f*_2_ is a maximum expected absolute deviation of a total controllable COD loading at each scenario from an expected total controllable COD loading; *f*_3_ is an expected sum of violated deviations of both COD and DO concentrations from the WQSs at loading points and/or stream junctions; and *f*_4_ is an expected sum of surplus deviations of both COD and DO concentrations from the WQSs at loading points and/or stream junctions. A prime objective is to minimize *f*_1_ to maximize the expected total controllable COD loading. Others are the functions to be minimized in order to generate noninferior solutions robust (i.e., less sensitive) against uncertainties of the input data or parameters.

Determining the coefficient matrices and right-hand side vectors of eq.[4] is important. Discharge from PS, *q^p^*, is usually known, while, discharge from NPS, *q^np^*, has to be estimated by some appropriate ways. The COD loading from NPS can be evaluated based on unit loading factors.

## RO and *ε*-RO models

In the RO framework, the Lagrangian method (a kind of the weighting method) is commonly employed to transform a vector optimization problem into a scalar one for obtaining noninferior solutions of the original problem. The single-objective model resulting from application of this method to the problem eqs.[3]–[10], hereinafter referred to as ‘RO model’, can be formulated as

[17] $$\text{Minimize }\;\;f_{1}\left(\cdot \right)+\alpha f_{2}\left(\cdot \right)+\beta f_{3}\left(\cdot \right)+\gamma f_{4}\left(\cdot \right)$$

subject to

[18] Constraints in eqs.[4]-[10]

where *α*, *β* and *γ* = weights which can vary from zero to infinity. When a noninferior solution can be obtained from the RO model, the following relations hold11)

[19] $$\alpha^{*}=-\frac{\partial f_{1}}{\partial f_{2}},\;\;\;\beta^{*}=-\frac{\partial f_{1}}{\partial f_{3}},\;\;\;\gamma^{*}=-\frac{\partial f_{1}}{\partial f_{4}},$$

where the superscript * denotes noninferior solution. This means that given weights in the RO model are identical to trade-off rates among objectives at the noninferior solution in objective space.

The alternative model formulation is to employ the *ε*-constraint method that only minimizes the prime objective function *f*_1_(·) in lieu of implementing eq.[17]. Maeda and Kawachi (2001)7) attempted to apply this method to the problem without consideration to wasteloads from nonpoint sources. When the method is used as a scheme for generating noninferior solutions to the multiobjective problem eqs.[3]–[10], the following so-called ‘*ε*-RO model’ is yielded.

[20] $$\text{Minimize }\;\;f_{1}\left(\cdot \right)$$

subject to

[21] Constraints in eqs.[4]-[10]

[22] $$f_{j}\left(\cdot \right)\leq {ɛ}_{j},\;j=2,3,4\;\;\;\left(ɛ-\text{constraints}\right)$$

where *ε_j_* = parameter associated with *j*-th objective criterion (*j* = 2, 3 and 4). Parametric variation of *ε_j_* in eq.[22] traces out a noninferior set (i.e., set of noninferior solutions) if the *ε*-constraints are binding, namely, *f_j_* = *ε_j_* holds. The *ε*-RO model produces the following trade-off rates among objectives, $\lambda_{1j}^{*}\;(j=2,3,4)$

[23] $$\lambda_{1j}^{*}=-\frac{\partial f_{1}}{\partial f_{j}},\;\;\;j=2,3,4$$

The *ε*-constraint *j* can be judged binding if the corresponding $\lambda_{1j}^{*}$ is found strictly positive.12) Otherwise the *ε*-RO model fails to produce noninferior solutions.

The weighting method generally fails to produce the entire noninferior set when it is non-convex, whereas the constraint method (e.g., the *ε*-constraint method) generates the whole noninferior set with any arbitrary shape.13) In addition, Cohon and Marks (1973)13) have insisted that the operational considerations of the constraint method makes it preferable to the weighting scheme, and have employed the constraint method in a water resource problem. The RO model (eqs.[17] and [18]) is a convex problem, and thus can produce a whole noninferior set. However, operating the *ε*-RO model (eqs.[20]–[22]) could be relatively effective compared to the RO model, because creating only useful, i.e., well reflecting decision-makers’ preference, alternatives by the RO model is difficult without setting optimal criteria in objective space. Furthermore, the RO model often fails to create various management options even if different weights in the synthesized objective functions are given. The RO model is indeed good at producing noninferior solutions, whether useful or useless to analysts or decision makers, being wasteful with computational time. Therefore it can be concluded that the *ε*-RO model is superior to the RO model with respect to efficient generation of alternatives worthy of discussion in decision-making.

## Optimization example

**Fig. 1:**
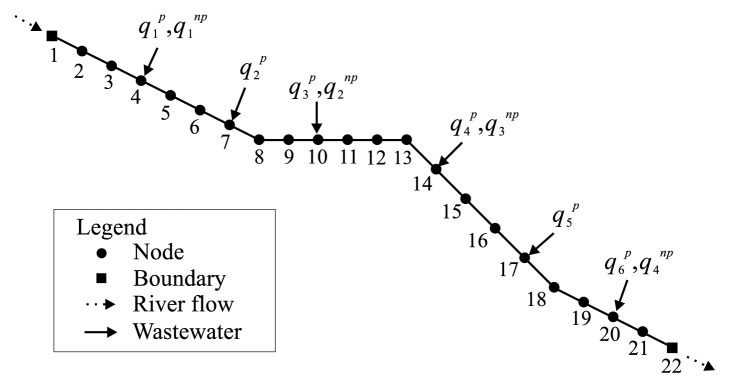
Hypothetical river.

A sample optimization by use of the *ε*-RO model is made for a hypothetical river system as shown in Fig. 1, which is 12.2 km long and 10m wide with uniform rectangular cross-sections. The river is discretized into 21 line elements with 22 nodes. The number of effluents from PSs and NPSs are six and four, respectively, whose injection points are also depicted in Fig. 1. Each discharge of wastewater released from PS, $q_{j}^{p}$, *j* =1*,. . .*,6 is shown in Table I. Land use in this river basin is categorized into paddy field, upland crop field, city and forest. The COD-based unit loading factors for individual land use types are assumed as in Table II. The NPS-born COD loads applied to the loading points (nodes) are listed in Table III including the areas of the respective land uses.

All kinds of constraints in the *ε*-RO model need to be determined under every scenario. Discrete values of the key parameters in twelve scenarios with their assumed probabilities of occurrence are listed in Table IV. Since there exist only one upstream boundary and one downstream boundary, the subscript ‘1’ on variables *Q^b^*, *L^b^*, *C^b^* and *h^b^* is dropped for simplicity. Using the values of *Q^b^* and *h^b^*, discharge, water depth and cross-sectional area in the main stream are first determined by solving the continuity and momentum equations

[24] $$\frac{dQ}{dx}-{\bar{q}}^{p}-{\bar{q}}^{np}=0,$$

[25] $$\frac{dF_{M}(h,Q)}{dx}+S_{M}(h,Q)=0,$$

where

$$\begin{array}{l} F_{M}=\frac{\xi Q^{2}}{A(h)}+g\int_{0}^{h}A(z)dz,\\ S_{M}=-g\int_{0}^{h}\frac{\partial A(z)}{\partial x}dz+gA(h)\frac{\partial z_{b}}{\partial x}+g\frac{n^{2}Q|Q|}{A(h)R^{4/3}}, \end{array}$$

*z* = vertical distance above a horizontal datum (m), *z_b_* = elevation of channel bed (m), *h* = water depth (m), *A* = cross-sectional area (m^2^), *g* = gravitational acceleration (m·s^−2^), *ξ* = velocity distribution coefficient, *n* = Manning’s roughness coefficient (s·m^−1^*^/^*^3^), and *R* = hydraulic radius (m). Here the values for *ξ* and *n* are assumed as 1 and 0.035, respectively. These equations [24] and [25] are solved by the FEM and finite volume method, respectively.10) Second, using these obtained hydraulic variables and the given data on water temperature, the parameters *D_x_*, *C_S_*, *K*_1_ and *K*_2_ are evaluated by

[26] $$D_{x}=mnR^{\frac{5}{6}}\frac{Q\sqrt{g}}{A}\;\;\;\left({\text{m}}^{2}\cdot {\text{s}}^{-1}\right),$$

[27] $$K_{1}=0.23{\left(1.047\right)}^{T-20}\;\;\;\left({\text{day}}^{-1}\right),$$

[28] $$K_{2}=\frac{3.9}{h^{\frac{3}{2}}}\sqrt{\frac{Q}{A}}{\left(1.024\right)}^{T-20}\;\;\;({\text{day}}^{-1}),$$

[29] $$C_{S}=1.43(10.291-0.2809T+0.006009T^{2}-0.0000632T^{3})\;\;\;(\text{mg}\cdot {\text{L}}^{-1}),$$

where *m* = undetermined parameter which varies within the range of 50 to 700 in natural streams, here taken as 50. Finally, taking the parametric values so obtained as known and specifying the upstream boundary values, *L^b^* and *C^b^*, as in Table IV, the FEM is again used to cast eqs.[1] and [2] into a system of linear algebraic equations.10) The equations system obtained is qualified as the constraints in eq.[4]. Effluent limitations are $L_{js}^{pl}=0$ and $L_{js}^{pu}=160\;\left(\text{mg}\cdot {\text{L}}^{-1}\right)$ in eq.[5], and WQSs for COD and DO concentrations are $L_{is}^{ou}=3.0\;\left(\text{mg}\cdot {\text{L}}^{-1}\right)$ and $C_{is}^{ol}=7.5\;\left(\text{mg}\cdot {\text{L}}^{-1}\right)$ in eqs.[6] and [7], respectively.

**Fig. 2:**
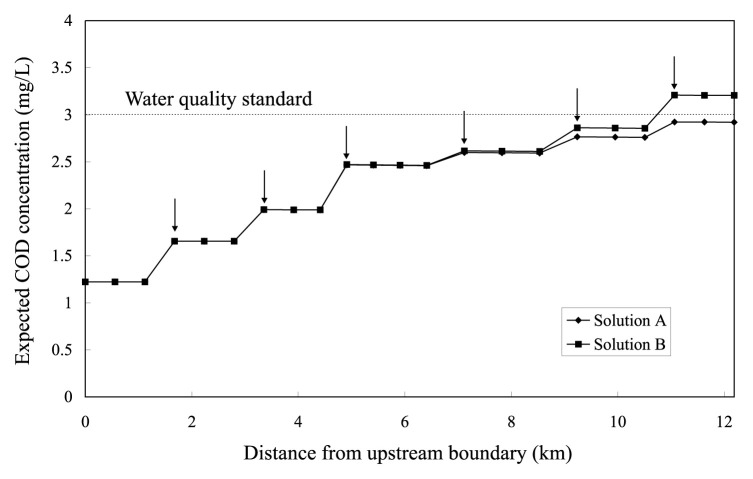
Expected COD concentration profiles (Vertical arrows indicate loading points).

**Fig. 3:**
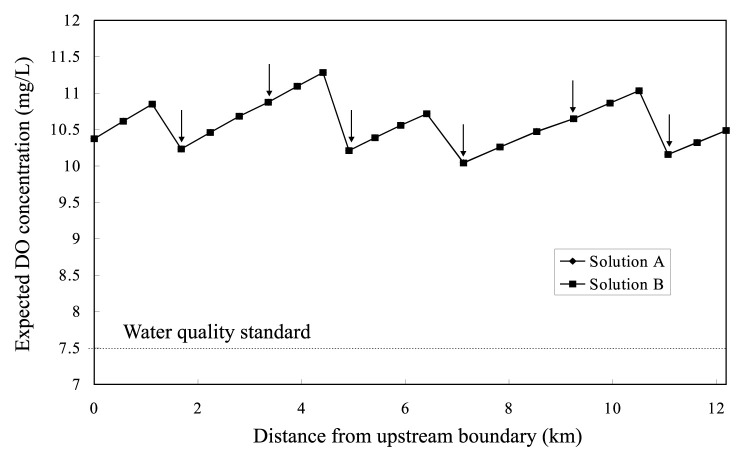
Expected DO concentration profiles (Vertical arrows indicate loading points).

The *ε*-RO model requires prior determination of values of the parameters *ε*_2_, *ε*_3_ and *ε*_4_ which bind the objective criteria *f*_2_, *f*_3_ and *f*_4_ in eq.[22], respectively. This determination is begun with finding minimum value of each criterion. For this, different three single-objective problems are solved that take the respective criteria as objective functions to be minimized under the constraints eqs.[4]–[10]. The resultant values of the functions minimized give the lowest limits of binding the criteria. Each of the criteria must therefore be bound to a *ε*-value larger than the corresponding lowest limit. In the present case, minimum values of the objective criteria *f*_2_, *f*_3_ and *f*_4_ are found to be 0 (g·s^−1^), 0 (mg·L^−1^) and 20.8613 (mg·L^−1^), respectively. Thus different two sets of *ε*-value, (*ε*_2_, *ε*_3_, *ε*_4_)=(0.3,0.1,20.8655) and (*ε*_2_, *ε*_3_, *ε*_4_)= (0.3, 0.5, 20.8645), are considered to produce sample noninferior Solutions A and B, respectively. Profiles of expected COD and DO concentrations along the river in Solutions A and B are shown in Figs. 2 and 3, respectively. Fig. 2 indicates that adopting Solution A as a management policy would keep the expected COD concentration lower than its standard value (3.0mg·L^−1^) along the whole stretch of the river. Meanwhile Solution B violates the COD standard at the downstream part of the river. This is a result of raising the allowable limit of violated deviation from *ε*_3_ =0.1 to *ε*_3_ =0.5. Indistinguishable difference can be seen between expected DO concentration profiles in Solutions A and B, shown in Fig. 3.

The respective results for optimal allocation of PS-born COD loadings at the six outfalls are shown in Tables V and VI including uncontrollable NPS-born loadings. From comparison of these tables, it can readily be seen that Solution B with increased COD loadings at nodes 10, 14, 17 and 20 allows more COD load into the river than Solution A by 1.5024 g·s^−1^. There exists a trade-off between increase of allowable COD load and compliance of the WQSs. Many management options other than Solutions A and B can also be obtained by the *ε*-RO model, which could assist in decision-making.

## Conclusions

An optimization model for allocating COD load has been developed within the RO framework, taking uncontrollable NPS waste loading into account. Inclusion of the COD loadings from paddy fields, upland crop fields, cities and forests in the optimization provides more persuasive management alternatives for better controlling water quality in rivers. The way of generating scenarios can be made more practical by considering monthly-based over-the-year uncertainties of discharge, water depth, COD and DO concentrations, water temperature and wastewater discharge from NPS. From both theoretical and practical aspects, the *ε*-RO model that employs the *ε*-constraint method to effectively and significantly constrain noninferior solutions could be a viable alternative to the conventional RO-model resorting to the Lagrangian method. Application of the *ε*-RO model to a hypothetical river exemplifies that it can provide various management options on COD load allocation achieving conflicting four objectives with preference of decision-makers.

Making no allowance for cost-effectiveness, public waters as in rivers or lakes should primarily be protected against their quality deterioration. In this context, the *ε*-RO model presently developed can serve as a master supervisory model, deciding effluent quotas to outfalls (or local dischargers like industries or sewage treatment plants) along the line of preference of decision-makers (or pollution control supervisors). The dischargers are then required to control pollutant emission below their given quotas. Using appropriate minor or local optimization models formulated in a cost/benefit sense, if need be, they can decide the cost-effective treatment levels or capacities while satisfying such a quota-related constraint as well.

## Figures and Tables

**Table I:** Discharge of wastewater released from PS

ID *j*	Node	Discharge$q_{j}^{p}\;({\text{m}}^{3}\cdot {\text{s}}^{-1})$

1	4	0.01
2	7	0.01
3	10	0.02
4	14	0.01
5	17	0.02
6	20	0.02

**Table II:** Unit loading factor

Land use	COD load (g·ha^−1^·day^−1^)

Paddy field	118.0
Upland crop field	62.0
City	144.0
Forest	51.4

**Table III:** Area of land use and COD load from NPS

Node	Paddy (m^2^)	Upland (m^2^)	City (m^2^)	Forest (m^2^)	Load (g·s^−1^)
4	8.0×10^5^	50	1.3×10^6^	9.6×10^6^	0.8970
10	6.0×10^5^	100	1.0×10^6^	8.0×10^6^	0.7245
14	8.0×10^5^	100	1.3×10^6^	1.0×10^7^	0.9208
20	5.0×10^4^	300	4.0×10^6^	6.0×10^4^	0.6768

**Table IV:** Key parameters of scenarios assumed in optimization

*s*	*Q^b^* (m^3^·s^−1^)	*h^b^* (m)	*L^b^* (mg·L^−1^)	*C^b^* (mg·L^−1^)	*T* (°C)	$q_{1}^{np}\;({\text{m}}^{3}\cdot {\text{s}}^{-1})$	$q_{2}^{np}\;({\text{m}}^{3}\cdot {\text{s}}^{-1})$	$q_{3}^{np}\;({\text{m}}^{3}\cdot {\text{s}}^{-1})$	$q_{4}^{np}\;({\text{m}}^{3}\cdot {\text{s}}^{-1})$	*p_s_*
1	3.0	1.01	1.1	12.5	5.0	0.3	0.5	0.4	0.5	31/365
2	4.0	1.13	1.2	12.3	4.0	0.3	0.5	0.4	0.5	28/365
3	10.0	1.80	1.0	11.6	6.0	0.5	0.7	0.6	0.7	31/365
4	6.0	1.39	1.2	10.7	10.0	0.4	0.6	0.5	0.6	30/365
5	6.4	1.43	2.0	10.0	15.0	0.4	0.6	0.5	0.6	31/365
6	4.0	1.13	1.8	9.7	19.0	0.3	0.5	0.4	0.5	30/365
7	11.0	1.89	1.6	8.6	22.0	0.5	0.7	0.6	0.7	31/365
8	4.0	1.13	1.3	9.0	25.0	0.3	0.5	0.4	0.5	31/365
9	5.0	1.24	1.2	9.1	23.0	0.3	0.5	0.4	0.5	30/365
10	9.0	1.72	0.8	9.3	18.0	0.5	0.7	0.6	0.7	31/365
11	4.0	1.13	0.9	10.8	14.0	0.3	0.5	0.4	0.5	30/365
12	7.0	1.49	0.6	11.0	8.0	0.4	0.6	0.5	0.6	31/365

**Table V:** Optimal allocation of COD load in Solution A

Node	Load from PS (g·s^−1^)	Load from NPS (g·s^−1^)	Total load (g·s^−1^)

4	1.6000	0.8970	2.4970
7	1.6000	0.0000	1.6000
10	3.0528	0.7245	3.7773
14	1.1868	0.9208	2.1076
17	1.4525	0.0000	1.4525
20	2.2339	0.6768	2.9107

Total	11.1260	3.2191	14.3451

**Table VI:** Optimal allocation of COD load in Solution B

Node	Load from PS (g·s^−1^)	Load from NPS (g·s^−1^)	Total load (g·s^−1^)

4	1.6000	0.8970	2.4970
7	1.6000	0.0000	1.6000
10	3.0619	0.7245	3.7864
14	1.2560	0.9208	2.1768
17	1.9106	0.0000	1.9106
20	3.1999	0.6768	3.8767

Total	12.6284	3.2191	15.8475
